# Supplementary material for: Molecular phylogeny of mulberries reconstructed from ITS and two cpDNA sequences
Source: PeerJ. 2019 Dec 12;7:e8158. doi: 10.7717/peerj.8158 (PMC6911693; doi:10.7717/peerj.8158)
Supplement: Supplemental Information 4 [file peerj-07-8158-s004.docx]

Table. S1 Detailed information used to identify ITS pseudogenes: GC content, minimum free energy of secondary structure, and conserved motifs in 5.8S rDNA

| Accessions | GC content | | | | | | | | | Minimum free energy | | | | | | | | | Conservative motifs(ITS-β) | | |
| --- | --- | --- | --- | --- | --- | --- | --- | --- | --- | --- | --- | --- | --- | --- | --- | --- | --- | --- | --- | --- | --- |
|  | ITS1 | | 5.8S | | ITS2 | | ITS | | ITS1 | | | 5.8S | | ITS2 | | ITS | | Motif1 | | Motif2 | Motif3 |
|  | α | β | α | β | α | β | α | β | α | | β | α | β | α | β | α | β |  |  |  |  |
| Agentingsang | 59.53 | 56.58 | 52.147 | 46.63 | 63.52 | 57.94 | 59.08 | 54.17 | -33.82 | | -32.01 | -20.10 | -19.44 | -43.73 | -31.94 | -98.87 | -85.52 | × | | √ | × |
| Baojing7 | 60.00 | 52.40 | 52.147 | 47.24 | 63.95 | 57.69 | 59.41 | 53.04 | -33.96 | | -26.20 | -20.10 | -16.60 | -42.30 | -28.62 | -97.58 | -73.00 | √ | | × | √ |
| Hanguodabaizhenzhu | 59.53 | 59.53 | 52.147 | 52.147 | 63.52 | 57.94 | 59.08 | 56.96 | -33.82 | | -33.82 | -20.10 | -19.44 | -43.73 | -33.60 | -98.87 | -91.18 | √ | | √ | √ |
| Huosang | 59.53 | 56.14 | 52.147 | 46.00 | 63.52 | 57.51 | 59.08 | 54.01 | -33.82 | | -32.01 | -20.10 | -19.44 | -43.73 | -31.94 | -98.87 | -85.28 | × | | √ | × |
| Jianpuzhai | 59.53 | 60.53 | 52.147 | 52.147 | 63.95 | 64.10 | 59.41 | 59.68 | -33.96 | | -37.48 | -20.10 | -18.25 | -42.30 | -44.73 | -97.58 | -101.68 | √ | | √ | √ |
| Lunjiao109 | 59.53 | 56.58 | 52.147 | 47.24 | 63.52 | 58.37 | 59.08 | 57.19 | -33.82 | | -35.29 | -20.10 | -19.44 | -43.73 | -33.89 | -98.87 | -94.21 | × | | √ | × |
| Mengsang | 59.53 | 59.53 | 52.147 | 52.147 | 63.52 | 63.95 | 59.08 | 59.25 | -33.82 | | -33.96 | -20.10 | -19.44 | -43.73 | -43.73 | -98.87 | -99.01 | √ | | √ | √ |
| Pisang2 | 60.00 | 56.28 | 52.147 | 48.47 | 63.95 | 62.66 | 59.41 | 56.63 | -33.96 | | -34.53 | -20.10 | -18.45 | -42.30 | -42.48 | -97.58 | -96.58 | √ | | √ | × |
| Shanxitiansang | 59.53 | 56.14 | 52.147 | 46.01 | 63.52 | 60.09 | 59.08 | 54.97 | -33.82 | | -35.53 | -20.10 | -17.89 | -43.73 | -37.81 | -98.87 | -92.82 | × | | √ | × |
| Shimiansang | 59.53 | 59.53 | 52.147 | 46.63 | 63.52 | 57.94 | 59.08 | 55.48 | -33.82 | | -33.82 | -20.10 | -19.44 | -43.73 | -31.94 | -98.87 | -86.87 | × | | √ | × |
| Xinyizhilai | 59.53 | 56.14 | 52.147 | 46.63 | 63.52 | 57.94 | 59.08 | 54.17 | -33.82 | | -32.01 | -20.10 | -19.44 | -43.73 | -31.94 | -98.87 | -85.52 | × | | √ | × |
| Yun6 | 60.00 | 60.53 | 52.147 | 52.76 | 63.95 | 64.10 | 59.41 | 59.84 | -33.96 | | -38.12 | -20.10 | -18.65 | -42.30 | -44.73 | -97.58 | -102.72 | √ | | √ | √ |
| Yun6muben | 60.53 | 57.02 | 52.76 | 49.69 | 64.10 | 61.37 | 59.84 | 56.73 | -37.48 | | -31.35 | -18.65 | -17.29 | -44.73 | -31.99 | -102.08 | -83.17 | √ | | √ | √ |
| Yun7 | 60.00 | 51.97 | 52.147 | 47.24 | 63.95 | 62.39 | 59.41 | 54.63 | -33.96 | | -27.15 | -20.10 | -15.97 | -42.30 | -35.95 | -97.58 | -80.50 | √ | | √ | √ |

ITS-α, α1, and β1 were placed in the column of α; ITS-α2, β, and β2 were placed in the column of β.
